# Does lumbar MRI predict degree of disability in patients with degenerative disc disease? A prospective cross-sectional study at University of Gondar comprehensive specialized hospital, North West Ethiopia, 2020

**DOI:** 10.1186/s12880-022-00866-7

**Published:** 2022-08-05

**Authors:** Yonathan Gebrewold, Bati Tesfaye

**Affiliations:** Department of Radiology, College of Medicine and Health Sciences, University of Gondar (UoG), Gondar, Ethiopia

**Keywords:** Lower back pain, Lumbar MRI, Oswestry Disability Index (ODI), Ethiopia

## Abstract

**Background:**

Low back pain (LBP) is one of the most serious public health problem globally with substantial socioeconomic implications. Degenerative disc disease is an important cause of LBP in the elderly. Magnetic resonance imaging (MRI) is routinely ordered by physicians in evaluation of patients with suspected degenerative disc disease in the lumbar spine. However there is no unanimous agreement in the literatures when it comes to the association of degree of disability to that of severity of lumbar MRI findings.

**Objective:**

The aim of this study is to assess the association between degree of disability measured using Oswestry Disability Index (ODI) and findings on lumbar spine MRI in patients with degenerative disc disease at University of Gondar comprehensive Specialized Hospital, North West Ethiopia, 2020.

**Methods and materials:**

A prospective cross-sectional study was conducted on 72 consecutively enrolled patients with degenerative disc disease who underwent lumbar MRI scan. Degree of disability was measured using ODI questionnaire translated to local language. Association between lumbar spine MRI parameters and ODI score and category was tested using Spearman’s rank correlation coefficient and Chi square tests.

**Results:**

The mean age of the study subjects was 43.81 ± 1.88 years (range 22–83 years). Forty-three (59.7%) of the study population were female. In terms of ODI category, most fell under minimal 33 (45.8%) or moderate 25 (34.7%) disability. Disc bulge (81.9%) and foraminal stenosis were the most frequent MRI abnormalities detected. ODI score showed weak correlation with grade of spinal canal stenosis. Grade of foraminal stenosis showed no correlation with ODI score.

**Conclusion:**

The clinical relevance of MRI findings in predicting degree of disability in patients with degenerative disc disease is limited and MRI study should be sparingly ordered in evaluation of these patients particularly in resource constrained settings.

**Supplementary Information:**

The online version contains supplementary material available at 10.1186/s12880-022-00866-7.

## Background

Lower back pain is a major public health problem globally with life time prevalence reaching 11–84% [1]. According to the Global Burden of Disease (GBD) report, over half a billion individuals across all age groups were affected by lower back pain in the year 2015. That figure is 17.3% larger compared to the prevalence in 2005. According to the same report LBP and neck pain are the leading global cause of disability in most countries [2]. In the United States acute LBP is the commonest cause of years lived with disability and third leading cause of disability adjusted life-years [3].

The clinical evaluation of LBP involves taking proper medical history, conducting a thorough physical examination and selection of relevant laboratory and imaging studies when indicated. The immediate goal of the evaluation process is early identification of potentially serious underlying cause and complications. A number of red flags are suggested in the literature to help clinicians not overlook serious underlying pathologies in patients with LBP. There is however lack of evidence to support the validity of the majority of those red flags used in clinical practice. A systematic review by Fillpo et al. has tried to identify and evaluate the most important red flags associated with LBP. The authors identified 26 red flags that raise suspicion for serious spinal disease like malignancy and spinal infection. Include in their list are advanced age, neurologic deficit, history of trauma, unexplained weight loss, fever and others. The presence of a combination of those red flags was diagnostically more accurate than the presence of those signs in isolation [4]. A more comprehensive and systematic framework to evaluation of LBP has been forwarded by the International Federation of Orthopaedic Manipulative Physical Therapists (IFOMPT). A team of experts lead by the IFOMPT built a decision tool for early diagnosis of serious spinal pathologies. The tool provided a three step process to identify four prioritized serious spinal pathologies, namely: cauda equina syndrome, vertebral fracture, malignancy and spinal infection. Accordingly, the clinical decision process begins by determining the level of concern considering the patients age, sex and presence of red flags, followed by decision on appropriate clinical action based on the level of concern and decision on the need for urgent referral [5]. Similarly, a joint clinical practice guideline from the American College of physicians and American Pain Society recommends classifying patients with LBP into one of three categories: Nonspecific LBP, back pain associated with radiculopathy or spinal stenosis and LBP associated with another specific spinal cause. The guideline discourages routine imaging or other diagnostic test in patients with nonspecific LBP and reserves the use of those diagnostic tests when severe or progressive neurologic deficit exists or when serious underlying conditions are suspected on the basis of clinical evaluation [6].

Numerous disease processes are incriminated in the development of chronic LBP. Mechanical causes including degenerative disc disease account for nearly 30% of the causes of chronic LBP [7]. Disc degeneration can be defined as an aberrant, cell mediated response to progressive structural failure or simply as a degenerated disc that is painful [8]. According to the lumbar disc nomenclature version 2.0 intervertebral disc disease is broadly classified in to degeneration and herniation. Degeneration include disc desiccation, disc space narrowing, disc bulge, mucinous degeneration, intradiscal gas and associated bone and marrow changes like endplate sclerosis and Modic changes. Herniation is defined as focal displacement of disc material involving less than 25% of the disc dimension on axial plane. A more diffuse disc material displacement is referred as disc bulge [9]. These disc changes are known to occur more frequently with increasing age [10, 11]. According to Boden et al. the prevalence of disc degeneration on at least one level was 35% and 100% in the age groups 20–39 and 60–80 years respectively [12]. Similarly Cheung et al. reported MRI detected degenerative disc changes in 40% of patients younger than 40 years and 90% of patients in the age group 50–55 years [13].

Imaging plays an important role in the diagnosis, pre-surgical evaluation and follow up of patients with LBP. The updated American College of Radiology (ACR) Appropriateness criteria recommends classifying patients into one of six variants in order to choose the appropriate imaging strategy. According to the guideline imaging is considered in patients who failed to respond to at least 6 weeks of medical or physical therapy and in patients with red flags [14]. Plain radiography, myelography, computed tomography (CT) and MRI have traditionally been used to identify morphological changes in the discovertebral unit. Recent advances in MRI have dramatically improved the ability to evaluate the spinal canal and neural structures with reasonable accuracy. Jung-Ha Kim et al. performed metaanalysis on diagnostic accuracy of MRI and CT in reference to surgical finding. The summary estimates of MRI sensitivity and specificity for surgically proven spinal abnormalities were 81.3% and 77.1% respectively [15].

Although MRI could provide ample information on the discovertebral status of patients with LBP, there are inconsistent reports in the literature on the ability of MRI parameters in predicting clinical severity. Freyr et al. based on their cohort of 109 consecutive patients reported that spinal canal stenosis measured on MRI correlated poorly with walking distance, level of leg and back pain, Oswestry Disability Index (ODI) and other measures of quality of life [16]. Vijay G Goni et al. found no correlation between ODI and anteroposterior diameter or cross-sectional area of the spinal canal [17]. Similarly, a retrospective review of 313 patients found no difference in rates of symptoms in MRI positive and MRI negative individuals. The same study however reported statistically significant difference in rate of surgery in the following year [18]. A prospective study of 200 individuals with baseline MRI, aimed to determine association of occurrence of new and serious lower LBP episodes and findings on follow up MRI taken around the time of pain revealed no difference in the baseline and follow up MRI [19]. On the other hand a systematic review of 14 studies (3097 subjects) reported that disc bulge (OR—7.5), spondylolysis (OR—5.1), disc extrusion (OR—4.4), disc protrusion (OR—2.7) and Modic 1change (OR—4) were more prevalent in patients with lower back pain compared to that of asymptomatic patients; whereas prevalence of disc extrusion, annular fissure, spondylolisthesis, Modic 2 & 3 changes and central canal stenosis were not statistically different between the two groups. The study however included individuals less than 50 years of age [20]. Arpinar et al. using dynamic contrast enhanced MRI reported significant correlation between end plate enhancement and degree of disability based on ODI [21].

Though there have been previous studies on patterns and prevalence of MRI finding from Africa, their clinical significance in terms of predicting level of pain and disability hasn’t been explored sufficiently. In this study we purpose to assess lumbar MRI patterns and their association with degree of disability measured using ODI index in a third world setting.

## Methods

### Study area

University of Gondar comprehensive specialized Hospital is located in Gondar town, North West part of Ethiopia, 738 km from Addis Ababa. Gondar town is the capital of Central Gondar zone of Amhara Region. The Hospital is a major tertiary teaching Hospital giving service to over five million population across the region.

The Department of Radiology provides diagnostic and basic interventional services. The department is equipped with two multi-detector CT scans (64 and 4 slice CT scans), one 1.5 T MRI, multiple multipurpose ultrasound machines and 2 digital radiography machines.

### Study design

Hospital based prospective cross sectional study design was employed for this study.

### Study period

The study was conducted between June and September 2020.

### Study population

*Study population* All patients above 20 years of age with suspected degenerative disc disease referred to the Department of Radiology, UoG CSH for a lumbar spine MRI scan are considered as the study population.

### Inclusion and exclusion criteria

- *Inclusion criteria* All patients above 20 years of age with MRI evidence of disc degeneration or herniation who consented to be part of the study were included.
- *Exclusion criteria* Patients with Previous lumbar spine operation, patients with non-degenerative cause of lower back pain and patients with contraindication for MRI or those unable to complete lumbar spine exam were excluded.

### Variables of the study

#### Dependent variable

Oswestry Disability Index (ODI) is the outcome variable. ODI is a questionnaire containing 10 sections with six statements in each sections. The statements are scored from 0 to 5 depending on degree of pain and disability. Functional impairments such as personal care, lifting, walking, sitting, standing, sleeping, sex life, social life, and travelling are assessed in the questionnaire. ODI is a simple, condition-specific, and preferred multidimensional tool because patients can easily comprehend the form. Patients with a score of 0–20% disability are considered minimally disabled, meaning patients can cope with most living activities. A score of 21–40% meant patients are classified as moderately disabled, and these patient experience more pain and difficulty with sitting, lifting, and standing. Travel and social life are more difficult, and personal care, sexual activity, and sleeping are not grossly affected. A score of 41–60% meant patients are considered severely disabled. Severely disabled patients have increased pain intensity that impacts routine functions. A score of 61–80% puts a patient in the category of disabled requiring positive intervention. Finally, a score of 81–100% refers to a patient who is bedridden [22].

#### Independent variables

Sociodemographic variables included age and sex. Lumbar spine MRI parameters including but not limited to intervertebral disc desiccation, bulge, protrusion, Modic change, spinal canal narrowing, neural foraminal narrowing and degree of narrowing were recorded for every patient. The highest grade of spinal canal and foraminal stenosis was taken for comparison with ODI. The grading and operational definitions used for the MRI parameters are described next.

Neural foraminal stenosis refers to the narrowing of the bony exit of the nerve root caused by a decrease in the height of an intervertebral disk, osteoarthritic changes in the facet joints, cephalad subluxation of the superior articular process of the inferior vertebra, and buckling of the ligamentum flavum or protrusion of the annulus fibrosus.

- *Grade 0* refers to normal neural foramen (normal dorsolateral border of the intervertebral disc, normal form of epidural fat, no significant ligamentum flavum hypertrophy or facet joint arthrosis or osteophytes from foraminal margin).
- *Grade 1* defined as mild foraminal stenosis with partial effacement of perineural fat and preserved nerve root.
- *Grade 2* defined as moderate foraminal stenosis perineural fat obliteration in both longitudinal and transverse plane without compression of the exiting nerve root.
- *Grade 3* referred to severe foraminal stenosis showing circumferential effacement of perineural fat and nerve root compression [23].

Spinal canal stenosis was graded from mild to severe as follows:s

- *Grade 0* No lumbar stenosis.
- *Grade 1* Mild stenosis with separation of all cauda equina.
- *Grade 2* Moderate stenosis with aggregation of some of the cauda equina.
- *Grade 3* Severe stenosis with none of the cauda equina separately visible [24].

Modic changes which refers to vertebral end plate signal changes as a result of degeneration was graded as:

- *Modic type 1* low signal intensity on T1WI and high on T2WI, representing fibrovascular tissue, inflammatory changes and edema.
- *Modic type 2* high signal intensity on T1WI and iso-intense/high on T2WI, representing bone marrow replacement by fat.
- *Modic type 3* low signal intensity on both T1WI and T2WI, representing reactive sclerosis**.**

Disc desiccation: is classified as:-

- *Focal disc desiccation* involvement of two or less than two discs.
- *Multifocal disc desiccation* involvement of greater than two discs.

### Sample size and sampling procedure

All eligible patients who presented to the Radiology department with in the study period were enrolled consecutively. A total of 72 patients who fulfilled the inclusion criteria were finally included in the study.

### Data collection procedure

All the lumbar spine MRI scans were taken using 1.5 T Philips Achieva MRI machine by experienced radiographic technicians. Axial and sagittal T1 & T2 weighted and sagittal STIR sequences were taken for the vertebral segments L1 to S1. No contrast was used for the MRI studies. All scanned spine MRI studies were reported by a senior general radiologist. A subset of 16 difficult cases were independently reported by another general radiologist of comparable experience. The interobserver reliability for severity of spinal and foraminal stenosis was calculated using Kappa statistics.

Data on degree of disability was obtained by interviewing patients using an ODI questionnaire translated to local language. A semi-structured questionnaire was used to document all the other relevant socio demographic and imaging variables.

### Data processing and analysis

Data was checked for completeness and cleaned before analysis. No missing data was identified in the dataset. Descriptive statistics is presented in the form of frequencies and percentages for categorical variables and summary statistics for continuous variables. The dependent variable (ODI) was tested for normality using the Shapiro–Wilk Test and showed a skewed distribution. Spearman's non-parametric correlation was applied to determine the relationship between MRI parameters and ODI score. Chi square test was applied when comparing ODI category and categorical independent variables. Statistical significance was assumed if calculated *p* value was below 0.05. Data entry and analysis was carried out using IBM SPSS Statistics for Windows, Version 19.0 (IBM Corp, Armonk, NY) and Stata, version 16 (Stata Corp) respectively.

### Ethical considerations

Ethical approval was obtained from School of Medicine Ethical Review Committee, College of Medicine and Health Sciences, University of Gondar. All necessary measures were taken to ensure the research is performed in accordance with the Declaration of Helsinki. Written informed consent was obtained from the study participants before the study. Participants were informed about the purpose of the research and that they have full right to refuse, withdraw or completely reject part or all of their participation in the study. Participants were also assured that their treatment and relation with the hospital and/or other organizations were not be influenced by their withdrawal from the study. Confidentiality were ensured using anonymous checklist and questionnaire. The study followed the Strengthening of Reporting of Observational Studies in Epidemiology (STROBE) guideline (Additional file 1).

## Results

### Sociodemographic and clinical characteristics

**Fig. 1:**
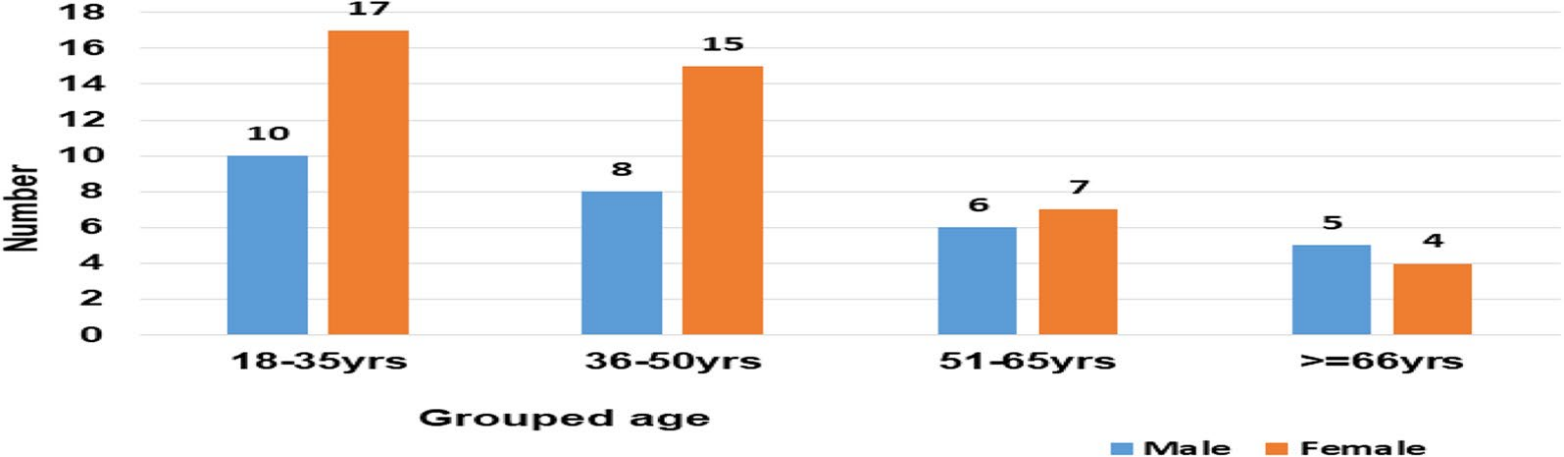
Distribution of cases on the basis of sex and age group

Out of the 72 participants 43 (59.7%) were female. Mean age of patients in this study was 43.8 ± 1.9 years (range 22–83 years). The median duration of lower back pain among participants was 2 years (IQR 0.5–4 years) (Fig. 1).

### MRI characteristics

Disc bulge (81.9%), foraminal stenosis (65.3%), disc desiccation (54.2%), Disc herniation (41.7%) and spinal canal stenosis (41.7%) were the most frequent MRI findings in our study in that order. Modic change was identified in 19.4% of the study subjects. L4/L5 and L5/S1 levels were where the above abnormalities most concentrated.

### Foraminal stenosis

Forty seven (65.3%) out of the total of 72 patients showed neural foraminal stenosis ranging from mild to severe stenosis. Mild neural foraminal stenosis was observed in thirty (41.7%), Moderate neural foraminal stenosis in ten (13.9%) and Severe stenosis in seven (9.7%) cases. Interobserver reliability measured using Kappa statistics was 0.65 suggesting moderate agreement between observers. With regard to level of foraminal stenosis L4/L5 was most frequently involved (65.3%) followed by L5/S1 and L3/L4 levels accounting for 31.9% and 22.2% respectively.

### Spinal canal stenosis

MRI scan of thirty patients (41.7%) demonstrated mild to severe degree of spinal canal stenosis. Mild, moderate and sever stenosis was observed in 13 (18.1%), 11 (15.3%) and 6 (8.3%) of patients respectively. Moderate interobserver agreement (kappa statistics—0.74) was observed between the two interpreters. Spinal canal stenosis was most frequent at L4/L5, L5/S1 and L3/L4 levels with frequency of 37.5%, 19.4% and 15.3% respectively.

### Disc bulge and disc herniation

Fifty (81.9%) of the study participants had disc bulge on MRI. L4–L5 disc was the most frequently involved (65.3%) followed by L5–S1 (50%) and L3–L4 (29.2). Thirty cases (41.7%) of the total 72 patients showed disc herniation. Central disc herniation was the commonest type observed in twenty one cases (29.2%); paracentral herniation was seen in nine cases (12.5%). Disc herniation was most frequently observed at L4/L5, L5/S1 and L3/L4 levels with a frequency distribution of eighteen (25%), thirteen (18.1%) and six (8.3%) respectively.

### Modic change

Modic change was present in 14 (19.4%) of the studies. Type II Modic change was the predominant type accounting for 86% (12 out of the fourteen). L3–L4 (11.1%) segment was the most frequently involved followed by L2/3 (4/14) and L4/5 (4/14). Summary of the MRI patterns and ODI categories is presented in Table 1.

**Table 1:** Frequency distribution of MRI parameters and ODI category

Variables	Freq	Percent (%)
Disc bulge	59	81.90
Foraminal stenosis	47	65.30
Grade 0	25	34.70
Grade 1	30	41.70
Grade 2	10	13.40
Grade 3	7	9.70
L1-L2	1	2.10
L2-L3	2	4.30
L3-L4	16	34
L4-L5	43	91.50
L5-S1	23	48.90
Spinal canal stenosis	30	41.70
Grade 0	42	58.30
Grade 1	13	18.10
Grade 2	11	15.30
Grade 3	6	8.30
L1–L2	1	3.30
L2–L3	1	3.30
L3L4	11	36.70
L4–L5	27	90
L5–S1	14	46.70
Disc herniation	30	41.70
Central	21	70
Paracentral	9	30
Modic Change	14	19.40
Type one	1	7.10
Type two	12	85.70
Type three	1	7.10
ODI GROUP		
minimal disability	33	45.80
Mod. disability	25	34.70
Severe disability	10	13.90
Crippled	3	4.20
bed ridden	1	1.40

### ODI index

The median score for the ODI index was 22 (IQR 14.5–37). In terms of ODI category, most (45.8%) fell under minimal disability. Moderate and severe disability was found in 34.7% and 3.9% of patients respectively.

Univariate analysis using Spearman’s rank non parametric correlation test revealed no statistically significant association between ODI score and individual MRI parameters except for grade of spinal canal stenosis. Spearman rank correlation coefficient (r) for Grade of spinal canal stenosis was 0.3 (*p* = 0.01). The association was not maintained when ODI score was categorized and compared with presence of spinal canal stenosis (chi square 4.6, *p* = 0.33). Grade of foraminal stenosis, disc desiccation, bulge, protrusion and Modic change showed no correlation with ODI score. (Table 2).

**Table 2:** Spearman correlation coefficient between MRI parameters and ODI index

Variable	r	*p* value
Grade foraminal stenosis	0.17	0.15
Grade of spinal canal stenosis	0.29	0.01
Disc bulge	0.138	0.247
Disc desiccation	0.237	0.054
Disc herniation	0.059	0.625
Modic change	− 0.07	0.556

## Discussion

Disc bulge and foraminal stenosis were the two most prevalent MRI findings among our study population, followed by disc desiccation, disc herniation and spinal canal stenosis. Most of the patients with foraminal or spinal canal stenosis had mild degree of stenosis and those pathologies tended to concentrate at L4/5 and L5/S1 levels. The prevalence of disc bulge (82%) and foraminal stenosis (65.3%) in the current study is relatively higher than previously reported. Brinjikji et al. in their meta-analysis estimated the prevalence of disc bulge to be 43.2%. Whereas a hospital based study from Ethiopia puts the prevalence much lower at 18.5%. On the other hand the distribution of spinal segment involvement in our study was consistent with findings from previous researches [20, 25].

Our study revealed a weak correlation between MRI grades of spinal canal stenosis and ODI score (r = 0.3). A spearman rank coefficient of 0 to 0.4 is considered a weak correlation in most grading systems [26]. Similar finding is reported in a study conducted in 1990 by Hurri et al. who reported association between spinal stenosis and ODI index [27]. However plain radiography instead of MRI was used to estimate spinal canal stenosis in that study and their finding couldn’t be substantiated by subsequent studies which tried to explore association between spinal canal area or anteroposterior diameter with that of ODI score. Sigmundsson et al. tried to study the relationship between objectively measured spinal canal stenosis and different measures of functional status and disability in 109 consecutive patients with spinal stenosis. They compared ODI score to that of the cross sectional area of the most narrowed dural sac and the number of disc levels with dural sac area < 70 mm^2^ and found no statistically significant correlation between those variables and ODI category [16]. Sirvanci et al., in their study on 63 patients with degenerative disc disease also reported no statistically significant association between degree of disability measured in ODI and dural sac cross-sectional area or severity of foraminal stenosis on MRI [28]. The same result is reproduced by Goni et al. who also failed to establish significant correlation between ODI score and anteroposterior diameter or cross sectional area of the spinal canal [17]. Even though our study demonstrated some correlation between grade of spinal canal stenosis on MRI and ODI score; the association couldn’t be maintained when Categorical ODI group, which is more clinically meaningful, instead of absolute ODI score is used for comparison. Further study is required to conclusively determine association between spinal canal stenosis and degree of disability. Grade of foraminal stenosis, disc bulge or herniation, Modic change and disc desiccation didn’t predict disability in the current study.

Multitude of hypothesis have been forwarded in the literatures for the reported lack of association between MRI parameters and clinical disability. Variation in what is a normal canal size throughout the population could be cited as one factor. Athiviraham et al. attempted to address this issue by adjusting the lumbar spinal canal narrowing on lumbar radiographs in reference to vertebral body size. They compared clinical disability measured in modified Roland-Morris score (RMS) to thecal sac diameter to vertebral body diameter and cross sectional area to vertebral body ratios. None of the adjusted spinal canal parameters were inversely correlated with RMS scores. They however noted greater functional disability in patients with severe spinal canal stenosis using 70 mm^2^ as a cut-off point [29]. The other explanation for the observed lack of association between MRI and disability parameters could be absence of universally accepted MRI grading system for foraminal and spinal canal stenosis. In addition, spinal canal narrowing is dynamic and changes with posture assumed by the patient. Therefore a measurement taken on MRI in supine position may not be predictive of patient’s symptom. And mere presence of spinal canal stenosis unless followed by compression of neural structures is unlikely to lead to clinical diseases [30].

Routine MRI investigation of patients with lower back pain for the sake of identifying spinal canal or foraminal stenosis therefore should not be encouraged. Previous studies also indicate that up to 41.5% of patients undergo lumbar MRI unnecessarily [18]. The treatment delay because of a long waiting list for an MRI study and the financial burden such unnecessary investigations incur are significant particularly in lower and middle income countries where MRI is not readily available.

### Limitations

Our study is not without limitations. Small sample size and relatively few cases in the category of severe canal stenosis could have limited the power of the study. The qualitative grading system used for staging spinal canal and foraminal stenosis may have introduced misclassification. We recommend that our study is interpreted in light of these limitations.

## Conclusion

This study tried to investigate the pattern of MRI abnormalities in adult patients with degenerative disc disease and determine their association with Oswestry disability score (ODI). Disc bulge and foraminal stenosis were the two commonest MRI patterns identified and their prevalence was relatively higher in the current study compared to previous reports. Grade of spinal canal stenosis is weakly correlated with ODI score where as foraminal stenosis and other discal MRI parameters showed no association with degree of disability. MRI has limited relevance when it comes to predicting degree of clinical disability in patients with degenerative disc disease and should be utilized judicially particularly in resource limited settings.

## Supplementary Information

**Additional file 1.** Supplemental material contains excerpts from patient Information sheet and questionnaire.

## Data Availability

All relevant data will be provided up on request to the corresponding author with the commitment to cite this work.

## Abbreviations

ACR: American College of Radiology
GBD: Global Burden of Disease
CT: Computed tomography
IFOMPT: International Federation of Orthopaedic Manipulative Physical Therapists
IVD: Intervertebral disc
LBP: Lower back pain
MRI: Magnetic resonance imaging
ODI: Oswestry Disability Index
OR: Odds ratio
r: Correlation coefficient
RMS: Roland-Morris score
STROBE: Strengthening of reporting of observational studies in epidemiology
T1WI: T1 Weighted image
T2WI: T2 Weighted image
UoG: University of Gondar
UoGCSH: University of Gondar Comprehensive Specialized Hospital
