# Supplementary material for: Does lumbar MRI predict degree of disability in patients with degenerative disc disease? A prospective cross-sectional study at University of Gondar comprehensive specialized hospital, North West Ethiopia, 2020
Source: BMC Med Imaging. 2022 Aug 5;22:138. doi: 10.1186/s12880-022-00866-7 (PMC9354276; doi:10.1186/s12880-022-00866-7)
Supplement: Supplementary file 1 — Additional file 1. Supplemental material contains excerpts from patient Information sheet and questionnaire. [file 12880_2022_866_MOESM1_ESM.pdf]

Supplementary material

Annex-I: Information sheet

Introduction

This information sheet and consent form is prepared with the aim of explaining the research project that you are asked to join by the group of research investigators. The main aim of the study is to assess MRI patterns in patients with degenerative disc disease and determine their correlation with degree of disability. The research group includes Radiologists and Residents from University of Gondar Hospital.

I invite you to take part in our project. If you think that this research is worth participating you will be asked to sign the consent form. Data collectors will review your chart or interview you. If you are reluctant to participate you will have the full right to withdraw.

All of your responses, your name and data taken from the chart are confidential by a coding system.

Risk and discomfort

You will be asked to come to radiology department on the day of appointment and after MRI scanning you will be asked questions related to your illness for 3 to 5 minutes. Your response will be documented.

Incentive

This MRI scanning (investigation) is valuable for carrying out study & help add some improvement in quality of care of patients in the future.

Confidentiality

Other than the investigators no one will have access to your collected data. Your name will not be documented in the questionnaire you fill. The data will be kept coded and locked with no labelling specifying you or anyone participating in the research.

Right to refuse

You have a full right of refusing at or during the conduct of the research. Your

Withdrawal will not affect other treatments and your relation with hospital.

For more information

If you want to get more information about the research and would like to give feedback, you can contact the investigator whose address is indicated below.

Yonathan Gebrewold, Department of Radiology, University of Gondar.

Phone:

Annex II Questionnaire (excerpt)

Part1: Socio-demographic data

S.N	Question	Possible answers	remark
01	Hospital card number	_______________	
02	Age	_____years	
03	Gender	1.male 2.female	

Part2: Factors related with clinical diagnosis

S.N	Questions	Possible answers	duration
01	Presenting chief complaint	____________	
02	Duration of back pain	_____________	
03	Back trauma		
04	Previous back surgery		
04	Other symptom/sign	____________	
05	Clinical diagnosis		

Oswestry Low Back Pain Disability Questionnaire

Instructions

This questionnaire has been designed to give us information as to how your back or leg pain is affecting your ability to manage in everyday life. Please answer by checking ONE box in each section for the statement which best applies to you. We realise you may consider that two or more statements in any one section apply but please just shade out the spot that indicates the statement which most clearly describes your problem.

Section 1 – Pain intensity

I have no pain at the moment

The pain is very mild at the moment

The pain is moderate at the moment

The pain is fairly severe at the moment

The pain is very severe at the moment

The pain is the worst imaginable at the moment

Section 2 – Personal care (washing, dressing etc)

I can look after myself normally without causing extra pain

I can look after myself normally but it causes extra pain

It is painful to look after myself and I am slow and careful

I need some help but manage most of my personal care

I need help every day in most aspects of self-care

I do not get dressed, I wash with difficulty and stay in bed

Section 3 – **Lifting**

I can lift heavy weights without extra pain

I can lift heavy weights but it gives extra pain

Pain prevents me from lifting heavy weights off the floor, but I can manage if they are conveniently placed eg. on a table

Pain prevents me from lifting heavy weights, but I can manage light to medium weights if they are conveniently positioned

I can lift very light weights

I cannot lift or carry anything at all

Section 4 – Walking*

Pain does not prevent me walking any distance

Pain prevents me from walking more than 1 mile

Pain prevents me from walking more than ½ mile

Pain prevents me from walking more than 100 yard

I can only walk using a stick or crutches

I am in bed most of the time

Section 5 – Sitting

I can sit in any chair as long as I like

I can only sit in my favourite chair as long as I like

Pain prevents me sitting more than one hour

Pain prevents me from sitting more than 30 minutes

Pain prevents me from sitting more than 10 minutes

Pain prevents me from sitting at all

Section 6 – Standing

I can stand as long as I want without extra pain

I can stand as long as I want but it gives me extra pain

Pain prevents me from standing for more than 1 hour

Pain prevents me from standing for more than 30 minutes

Pain prevents me from standing for more than 10 minutes

Pain prevents me from standing at all

Section 7 – Sleeping

My sleep is never disturbed by pain

My sleep is occasionally disturbed by pain

Because of pain I have less than 6 hours sleep

Because of pain I have less than 4 hours sleep

Because of pain I have less than 2 hours sleep

Pain prevents me from sleeping at all

Section 8 – Sex life (if applicable)

My sex life is normal and causes no extra pain

My sex life is normal but causes some extra pain

My sex life is nearly normal but is very painful

My sex life is severely restricted by pain

My sex life is nearly absent because of pain

Pain prevents any sex life at all

Section 9 – Social life

My social life is normal and gives me no extra pain

My social life is normal but increases the degree of pain

Pain has no significant effect on my social life apart from limiting my more energetic interests eg, sport

Pain has restricted my social life and I do not go out as often

Pain has restricted my social life to my home

I have no social life because of pain

Section 10 – Travelling

I can travel anywhere without pain

I can travel anywhere but it gives me extra pain

Pain is bad but I manage journeys over two hours

Pain restricts me to journeys of less than one hour

Pain restricts me to short necessary journeys under 30 minutes

Pain prevents me from travelling except to receive treatment
